# Changes of serofast status in HIV negative asymptomatic neurosyphilis patients after treatment

**DOI:** 10.3389/fmed.2022.938016

**Published:** 2022-08-03

**Authors:** Jing Liu, Tian-Wei Zhao, Chun Zhou, Hui-Wen Yan, Wen-Hui Lun

**Affiliations:** ^1^Department of Dermatology and Venereology, Beijing Ditan Hospital, Capital Medical University, Beijing, China; ^2^Department of Clinical Laboratory, Beijing Ditan Hospital, Capital Medical University, Beijing, China

**Keywords:** syphilis, serofast, asymptomatic neurosyphilis, serological cure, treatment

## Abstract

Serofast status after therapy in syphilis patients is a common phenomenon. A proportion of patients who have serofast status exhibit abnormal cerebrospinal fluid test results, which can be defined as asymptomatic neurosyphilis (ANS); however, it remains unclear whether ANS patients can achieve serological cure after anti-neurosyphilis treatment as quickly as other serofast patients. In this study, non-treponemal pallidum antibody serological responses were studied in ANS and serofast control patients, and the cumulative rates of serological cure in the ANS group were 9.6, 22.1, 25.9, and 30.2% in 3, 6, 9, and 12 month after treatment, which were statistically higher than those of the serofast control group. The change gap in serological cure rates was even more pronounced within 6 months after treatment, but the majority of ANS patients had no change in serofast status at 12 months after treatment. Our study indicates that anti-neurosyphilis therapy can partially change the serofast status. As serofast status cannot easily be changed even under neurosyphilis treatment in the majority of patients, the pathogenesis of this condition needs further research.

## Introduction

Syphilis is an old disease that can cause serious health problems without treatment. Since the introduction of penicillin in 1943, there have been great improvements in syphilis treatment. Theoretically, syphilis is a sexually transmitted disease that can be cured. However, in actual clinical settings, judging whether syphilis has already been cured is not an easy task (1). Non-treponemal serological response is an important laboratory indicator of whether treatment has been effective, and serological cure is defined as either a negative rapid plasma regain (RPR) or ≥ 4-fold decrease (twofold dilution) in titer at 6–12 months following therapy. However, approximately 15–20% of patients with early syphilis do not meet the criteria of serological cure and are therefore referred to as being “serofast.” Serofast status is defined as a < 4-fold (twofold dilution) decline in non-treponemal antibody titer at 6–12 months or as persistently low titer after treatment (2). The etiology and the optimal management of serofast status remain unclear.

Retreatment is recommended for serofast patients if follow-up cannot be ensured, which is common in clinical practice. Several studies have reported no incremental benefit for re-treating HIV-negative serofast patients (3, 4). In our previous study, a subset of serofast patients exhibited cerebrospinal fluid (CSF) abnormalities that could be defined as asymptomatic neurosyphilis (ANS) according to the appropriate guidelines (5). Neurosyphilis is caused by *Treponema pallidum* infecting the central nervous system, which can occur at any stage of syphilis. ANS is a type of neurosyphilis without any neurological symptoms, and its diagnosis relies on CSF test abnormalities, including positive venereal disease laboratory research tests (VDRL), abnormal white blood cell (WBC) counts, and elevated protein (6). At present, disputation surrounds ANS, and thus, defining ANS has been extremely difficult and controversial (2). Most definitions rely on a combination of CSF laboratory tests (such as VDRL, WBC count, and protein level), but no consensus definition exists. Some research has suggested that a negative CSF fluorescent treponemal antibody absorption (FTA-ABS) IgG test as the exclusion criterion for neurosyphilis, but this is not unanimously recognized (7).

There are many different views about ANS. On the one hand, ANS is the early stage of neurosyphilis, which can develop into symptomatic neurosyphilis; therefore, ANS should be treated in accordance with neurosyphilis. On the other hand, it is thought that abnormal changes in CSF are common in the early stages of syphilitic infection, but most patients heal naturally without intervention. Thus, debate continues as to whether asymptomatic patients need CSF testing, but there is not enough evidence to determine whether the diagnosis and treatment of ANS could contribute to prognosis in these patients.

The aim of this study was to compare changes in non-treponemal antibody serum titer in ANS patients with those in serofast patients. In addition, the study evaluated whether anti-neurosyphilis treatment provides benefit for serological cure in these patients.

## Materials and methods

All patients in this study were enrolled from the clinical database of Beijing Ditan Hospital, Capital Medical University, between September 2015 and August 2018. All of the enrolled patients were HIV-negative and in serofast status. Serofast status was defined as described in the previous studies (5, 8, 9), as follows: (1) in early syphilis patients who were at least 6 months after initial recommended treatment and regular follow-up, no fourfold decline in serum RPR titer was observed even after additional treatment; (2) RPR titer exhibited a < 4-fold decline more than 1 year after treatment in late latent syphilis; and (3) patients who had a fourfold decline in serum RPR titer after initial treatment but exhibited no seroreversion or decline in non-treponemal antibody titer for more than 1 year. Lumbar puncture and CSF tests were carried out, and ANS was diagnosed as described in our previous study (5), which followed the European Guidelines on the Management of Syphilis and US CDC guidelines (2, 8). ANS was characterized by reactive CSF RPR or negative CSF RPR but CSF WBC count > 5 × 10^6^/L, or negative CSF RPR, CSF WBC count ≤ 5 × 10^6^/L but CSF protein concentration > 45 mg/dL, and without any neurological signs or symptoms. Other neurological diseases were excluded based on clinical history and physical examination.

According to the CSF examination results, serofast patients were divided into two groups: serofast patients with abnormal CSF test results were classified as the ANS group and the serofast patients with normal CSF test results were classified as the serofast control group. The ANS group was treated according to the recommended neurosyphilis treatment regime (8), and the serofast control group was followed up without recommended treatment. All patients were followed every 3 months for serological RPR card test. Serologically defined treatment response was used to classify the patients as serological cure (defined as patients whose RPR titer dropped > 4-fold or RPR became negative), non-response (RPR tier was not changed; drop or increase < 4-fold), or treatment failure (RPR titer increased > 4-fold) (9, 10).

The study was approved by the Institutional Ethics Committee of Beijing Ditan Hospital, Capital Medical University.

### Data analysis and statistics

Data were analyzed using IBM SPSS version 19.0. Figures were drawn using GraphPad Prism 8. Continuous variables are described using median and interquartile range (IQR), whereas categorical variables are described by numbers and percentages. Associations between categorical variables were assessed using the chi-square test. Titers were obtained 3, 6, 9, and 12 months after enrollment, and Kaplan–Meier product-limit survival curves were used to examine the rates of serologic response to treatment. All hypothesis testing was two-sided, and *P* < 0.05 was considered to be statistically significant.

## Results

### Baseline demographics and patient characteristics

According to the recommended CSF assessment criteria for asymptomatic serofast patients, 445 HIV-negative serofast syphilis patients were enrolled in Beijing Ditan Hospital, Capital Medical University, between September 2015 and August 2018. All of the patients had received lumbar puncture for CSF testing, and the median time from first diagnosis of syphilis to lumbar puncture was 24 (IQR 12, 36) months. Through CSF examination, 136 participants (30.6%) exhibited an abnormal CSF test that could be diagnosed as ANS (ANS group), whereas 309 participants (69.4%) exhibited a normal CSF test result (serofast control group). The demographic and clinical characteristics of the two groups are listed in Table 1. The baseline data for the ANS and serofast control groups did not exhibit any statistically significant differences in sex, duration of serofast status, initial stage of syphilis, initial serum RPR titer, or initial treatment regimen. The median age of the participants in the ANS group was greater than that of patients in the serofast control group (*P* = 0.023). The serum RPR titer of the ANS group was higher than that of the serofast control group at the time of lumbar puncture (Wilcoxon symbol rank and test, *P* = 0.013).

**TABLE 1:** Demographic and clinical characteristics of the ANS and the serofast control groups.

		ANS (*n* = 136)	Serofast control (*n* = 309)	*P*-value
Gender	Male (*n*, %)	57 (41.9)	100 (32.4)	0.052
	Female (*n*, %)	79 (58.1)	209 (67.6)	
Age (years, median and interquartile range)		36 (28, 52)	32 (27, 43)	0.023
Disease course (months, median and quartile)		24 (12, 36)	24 (12, 36)	0.615
Syphilis stage	Latent	115 (84.6)	267 (86.4)	0.697
	Primary	1 (0.7)	4 (1.3)	
	Secondary	20 (14.7)	38 (12.3)	
RPR titer (median and interquartile range)	≦ 1:2 1:4 1:8 1:16 ≧ 1:32	1:8 (1:4, 1:16) 18 (13.2) 28 (20.6) 42 (30.9) 26 (19.1) 22 (16.2)	1:8 (1:4, 1:16) 67 (21.7) 79 (25.6) 71 (23.0) 57 (18.4) 35 (11.3)	0.013
Initial regimen with benzathine Penicillin (*n*, %)		120 (88.2)	272 (88.0)	0.950
CSF RPR	Negative	118 (86.7)	309 (100)	NA
	1:1	11 (8.1)	–	
	1:2	5 (3.7)	–	
	1:4	2 (1.5)	–	
CSF WBC count/μL (*n*, %)	≤ 5	28 (20.6)	309 (100)	NA
	> 5	108 (79.4)	–	
CSF protein concentration mg/dL (*n*, %)	≤ 45 > 45	94 (69.1) 42 (30.9)	309 (100)	NA

NA, not available.

### Treatment and serological response

The patients in the ANS group were treated with aqueous crystalline penicillin G, 4 million units IV every 4 h for 14 days, followed by benzathine penicillin G, 2.4 million units IM weekly for 3 weeks. Re-treatment was not recommended in the serofast control group. All of the participants were followed every 3 months for serological tests.

The proportion of evaluable participants who exhibited serological response varied by group and time after therapy. The rates of serological cure at 3, 6, 9, and 12 months are shown in Table 2, the proportion of patients with serological cure in the ANS group was higher than that in the serofast control group and the difference was statistically significant between the two groups.

**TABLE 2:** Comparison of serological responses between the ANS group and the serofast control group.

		ANS (*n*, %) (*n* = 136)	Serofast control (*n*, %) (*n* = 309)	*P*-value
3 Months	Serological cure	13 (9.6)	13 (4.2)	0.027
	Serofast	122 (89.7)	293 (94.8)	0.047
	Treatment failure	1 (0.7)	3 (1.0)	1*
6 months	Serological cure	30 (22.1)	39 (12.6)	0.011
	Serofast	104 (76.4)	270 (87.4)	0.004
	Treatment failure	2 (1.5)	0 (0)	0.093*
9 months	Serological cure	35^#^ (25.9)	52 (16.8)	0.029
	Serofast	98^#^ (72.6)	255 (82.6)	0.012
	Treatment failure	2^#^ (1.5)	2 (0.6)	0.589*
12 months	Serological cure	41 (30.2)	62 (20.1)	0.020
	Serofast	94 (69.1)	244 (79.0)	0.025
	Treatment failure	1 (0.7)	3 (0.9)	1*

*Fisher’s exact test; ^#^n = 135.

### Time-dependent proportion of serofast patients

**FIGURE 1:**
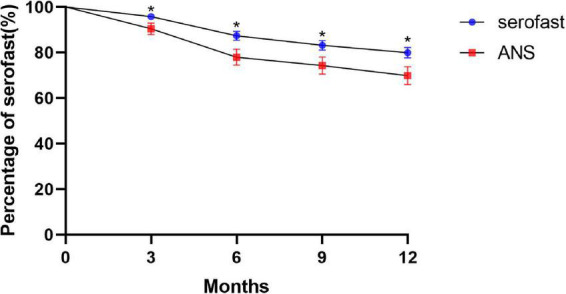
Kaplan–Meier plots of the number of patients remaining in serofast status in the ANS group after therapy as compared with the serofast control group (*P < 0.05).

Survival analyses using the log-rank test were conducted to determine the proportion of patients with unchanged serofast status. The time-dependent accumulated serological cure rates after treatment at 3, 6, 9, and 12 months are shown in Figure 1. The trend of the change in serological cure rates in both groups as shown in the talbe2 indicates that there was a significant difference between ANS and serofast control group in the serological cure rate at each follow-up time point, majority of serological cure cases appeared in the half year after treatment.

### Serological cure rates based on exhibited cerebrospinal fluid fluorescent treponemal antibody absorption

Some researchers have suggested CSF FTA-ABS as an exclusion criterion for neurosyphilis diagnosis, but others disagree (7). We tested the CSF FTA-ABS IgG and IgM titers in the ANS group. All patients were negative for CSF FTA-ABS IgM, but 75 patients were positive for CSF FTA-ABS IgG and 61 were negative for CSF FTA-ABS IgG. The serological responses between the two groups were compared.

**FIGURE 2:**
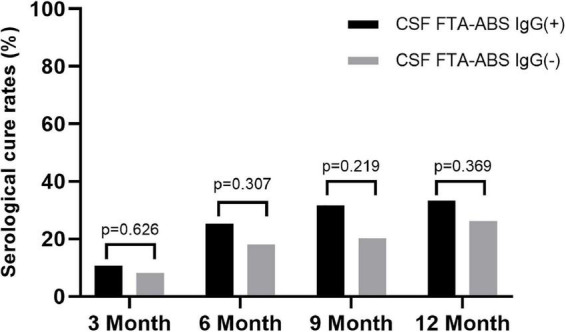
Serological cure rates based on CSF FTA-ABS in ANS group.

The rates of serological cure were 10.7, 25.3, 30.7, and 33.3% in the CSF FTA-ABS IgG(+) patients at 3, 6, 9, and 12 months, respectively. In contrast, the rates of serological cure were 8.2, 18, 21.3, and 26.2% at 3, 6, 9, and 12 months, respectively, in the CSF FTA-ABS IgG(-) patients. The difference in serological cure rates between the two groups was not significant (Figure 2).

## Discussion

Despite the availability of effective treatments, 15–20% of persons with early syphilis are referred to as being serofast, and the incidence of serofast status in late syphilis is much higher (1–4). At present, the etiology of serofast status remains unknown. Many studies have shown that re-treatment does not significantly improve a patient’s serological cure rate, and in serofast patients with early syphilis, it may not provide any significant benefit, even though re-treatment is commonly performed in clinical practice (3, 4). In our previous study, abnormal CSF test results were observed in a substantial proportion of serofast patients, which could be defined as ANS (5). CSF assessment is recommended in asymptomatic patients who experienced serological failure or are serofast (2). To our knowledge, there are no reports that describe the changes in non-treponemal serological titer in ANS patients after treatment.

In the present study, 136 serofast patients with abnormal CSF tests were treated according to the guideline for neurosyphilis therapy. As expected, a decline in RPR titer was observed as early as 3 months after treatment as compared with the control group. The rates of serologic cure gradually increased over time, from 9.6% in the third month to 22.1, 25.9, and 30.2% at 6, 9, and 12 months, respectively. A significant difference in serological cure rates as compared with the control group was observed. Our results demonstrate the benefit of serological cure in ANS patients after treatment and indicate the importance of screening for ANS in serofast patients. Treatment with a neurosyphilis-directed regimen could significantly improve the serological cure rate in this group of patients.

Some research has suggested that CSF FTA-ABS might be better suited to exclude rather than confirm a diagnosis of neurosyphilis, but there are limitations to CSF treponemal testing, as the negative predictive value of any test is dependent on the prevalence of the condition in the population in which the test is undertaken (11). Therefore, in a patient with a high pre-test probability, a negative CSF FTA-ABS result cannot exclude the diagnosis with sufficient confidence. It was observed that a negative CSF FTA-ABS is not sufficient evidence for clinicians to decide not to treat for neurosyphilis (7). We were therefore interested to determine if there were any differences in the rates of serological cure after treatment between CSF FTA-ABS IgG(+) and FTA-ABS IgG(–) ANS patients in our study. Interestingly, the serological cure rates in the CSF FTA-ABS IgG(–) group were lower than those in the CSF FTA-ABS IgG(+) group, but the differences were not statistically significant. In both the CSF FTA-ABS IgG(+) and CSF FTA-ABS IgG(–) groups, the serological cure rates were higher than those in the serofast control group. Indeed, CSF testing was repeated in 47 of the ANS patients after 6 months. Even though all of those patients had received treatment for neurosyphilis, the CSF RPR for one of the patients turned from negative to positive (titer of 1:1). In addition, 5 patients’ CSF FTA-ABS IgG results turned from negative to positive (data not shown). It is possible that a negative CSF FTA -ABS IgG test does not mean that the CSF FTA-ABS IgG test will always be negative in the future. Therefore, in the setting of CSF abnormalities, the role of a negative CSF FTA-ABS test in neurosyphilis diagnosis needs further investigation.

The survival curve chart clearly shows that the increase in the serological cure rate of syphilis is more obvious after 3 months and 6 months of neurosyphilis treatment, and the gap in the syphilis serological cure rate trend between the ANS group and the serofast control group became steady after 6 months. Although the difference in serological cure rates between the ANS group and serofast control group with neurosyphilis treatment was statistically significant, nearly 70% of cases in the ANS group retained serofast status at 12 months after treatment. In our previous study, the neuron damage biomarker of symptomatic neurosyphilis was not present in the majority of patients with ANS (12). This indicated that ANS only partly explains the serofast status. As the serofast status in the majority of patients cannot easily be changed even with neurosyphilis treatment, the pathogenesis of serofast status requires further research.

In this study, all of the participant have experienced secondary retreatment and at least one course of benzathine penicillin G, 2.4 million units IM weekly for 1–3 weeks, actually nearly 90% patients have received benzathine penicillin G anti-syphilis treatment in the initial regime and others in the secondary retreatment. So all of patients have enough treatment when they are diagnosed to be serofast status. because many studies have demonstrated that There is no evidence that patients will benefit from multiple retreatments in spite of this being commonly performed in clinical practice (3, 4), the serofast control group did not re-treat after CSF tests.

The present study has several limitations. First, this was a retrospective cohort study. CSF testing was repeated in only 34.6% (47 of 136) of patients in the 6 months after anti-neurosyphilis treatment in the ANS group. Good adherence to CSF testing during long-term follow-up is difficult in actual clinical settings. Although all ANS patients had undergone a full course of anti-neurosyphilis treatment, we could not determine the percentage of ANS patients in which the CSF returned to normal; Second, Although there was no statistical difference in the serological cure rate between the CSF FTA-ABS(+) and CSF FTA-ABS (-) groups, there was a difference in the serological cure rate between the two groups, which needs to be further studied with an enlarged sample size in the future. Third, there were some difference in baseline age and RPR titers between ANS and serofast control groups, which might reflected the facts in real world, we don’t think these difference will influence the results, Since the inclusion and exclusion criteria were the same, the indicator for evaluating the treatment effect was the change in RPR titer before and after treatment, rather than a direct comparison of RPR titers. Some studies suggest that the RRP titer declines more slowly in the elderly, but in this study, although there is a difference in age between the two groups, the older patients are in the ANS group, which may affect the serum cure rate of some ANS patients, but The statistical results of the comparison between the two groups were not affected.

## Conclusion

ANS is one of reasons for serofast status of syphilis patient, this study demonstrated that some ANS could achieve serological cure after anti-neurosyphilis treatment, However, a significant proportion of ANS remained serofast status after 1 year of treatment, and the reasons deserve further investigation.

## Data availability statement

The original contributions presented in the study are included in the article/supplementary material, further inquiries can be directed to the corresponding author/s.

## Ethics statement

The studies involving human participants were reviewed and approved by the Institutional Ethics Committee of Beijing Ditan Hospital, Capital Medical University. The patients/participants provided their written informed consent to participate in this study.

## Author contributions

JL and W-HL wrote the main manuscript text. T-WZ, CZ, and H-WY collected the clinical data. All authors contributed to the article and approved the submitted version.
